# Oral immunization with recombinant enterovirus 71 VP1 formulated with chitosan protects mice against lethal challenge

**DOI:** 10.1186/1743-422X-11-80

**Published:** 2014-05-06

**Authors:** Fushun Zhang, Chunsheng Hao, Shuo Zhang, Aqian Li, Quanfu Zhang, Wei Wu, Lin Liu, Chuan Li, Mifang Liang, Xiuling Li, Dexin Li

**Affiliations:** 1 Key laboratory of Medical Virology, NHFPC; Department of Viral Hemorrhagic Fever, National Institute for Viral Disease Control and Prevention, China CDC, Beijing 102206, China; 2 Beijing Institute of Biological Products Co. Ltd, Beijing 101111, China

**Keywords:** Enterovirus 71, VP1, Oral immunization, Virus challenge

## Abstract

**Background:**

Enterovirus 71 (EV71) is the etiologic agent of hand-foot-and-mouth disease (HFMD) in the Asia-Pacific region, Many strategies have been applied to develop EV71 vaccines but no vaccines are currently available. Mucosal immunization of the VP1, a major immunogenic capsid protein of EV71, may be an alternative way to prevent EV71 infection.

**Results:**

In this study, mucosal immunogenicity and protect function of recombinant VP1 protein (rVP1) in formulation with chitosan were tested and assessed in female ICR mouse model. The results showed that the oral immunization with rVP1 induced VP1-specific IgA antibodies in intestine, feces, vagina, and the respiratory tract and serum-specific IgG and neutralization antibodies in vaccinated mice. Splenocytes from rVP1-immunized mice induced high levels of Th1 (cytokine IFN-γ), Th2 (cytokine IL-4) and Th3 (cytokine TGF-β) type immune responses after stimulation. Moreover, rVP1-immunized mother mice conferred protection (survival rate up to 30%) on neonatal mice against a lethal challenge of 10^3^ plaque-forming units (PFU) EV71.

**Conclusions:**

These data indicated that oral immunization with rVP1 in formulation with chitosan was effective in inducing broad-spectrum immune responses and might be a promising subunit vaccine candidate for preventing EV71 infection.

## Background

Enterovirus 71 (EV71), a positive-sense, single-stranded RNA virus in the genus *Enterovirus*, family *Picornaviridae*, is associated with a spectrum of diseases, including HFMD, herpangina, encephalitis, aseptic meningitis, cerebellar ataxia, poliomyelitis-like syndrome and even some fatal diseases. There were several outbreaks of EV71 infection over the last ten years in Taiwan and mainland of China [1,2], and the infectious diseases caused by EV71 infection have become a serious public health threats in Asia, in particular to children.

A wide range of strategies have been applied to develop EV71 vaccines, including attenuated live vaccine [3,4], inactivated vaccine [5,6], Virus-like particles (VLP) vaccine [7], VP1 subunit [6] or DNA vaccine [6,8], et al. and their efficacy has been studied in animals or even clinical trials. However, most of the vaccines mentioned above were delivered by injection which was general more costly, difficult to administrated, and less acceptable to children. Mucosal immunizations, including oral, intranasal, pulmonary, rectal and vaginal routes of vaccine administration, stimulate a stronger mucosal immune response that could protect mucous membranes against microbial colonization and invasion. Oral vaccination is a desirable alternative, potentially allowing for safe, simple, rapid delivery and takes advantage of the protection offered by intestinal mucosa [9]. To potentiate mucosal immune responses and protect antigens from the harsh environment of the alimentary canal, adjuvant is usually required. Previous studies showed that chitosan, as a potential use non-toxic delivery component, boosts both the humoral and cellular immune responses of the mucosal surface after co-administration with *Helicobacter Pylori* and influenza vaccines [10,11] and causes little irritability or antigenicity.

The capsid of EV71 is comprised of 60 identical protomers, each of which contains a copy of four viral structural proteins VP1-VP4. VP1 has been defined as a major neutralizing antigen which effectively induces immune protections against EV71 infection, has been considered as potential subunit vaccine. Previous studies identified major linear neutralizing epitopes and CD4^+^ T-cell epitopes within the VP1 protein [12,13], suggesting it is a promising vaccine candidate. In this study, the mucosal immunogenicity and protective function of *Escherichia coli* (*E. coli*) expressed rVP1 protein in formulation with chitosan were tested in female ICR mice and the protective efficiency of the rVP1 immunogen was evaluated in vitro and in vivo.

## Materials and methods

### Purification of EV71 Virions

Vero cells (ATCC CCL81) were cultured in Dulbecco’s modified Eagle’s medium (DMEM; Gibco) with 10% heat-inactivated fetal bovine serum (FBS), penicillin and streptomycin (PS; 100U/ml) at 37°C with 5% CO_2_. EV71 strain (HN08) (Genebank No. HM038013) used in this study was isolated in Henan province and was propagated in Vero cells cultured in DMEM containing 2% FBS and PS (100 U/ml). When 90% of cells exhibited cytopathic effect (CPE), supernatants were harvested and inactivated with β-propiolactone (1:2000). Crude supernatants were centrifuged at 12,000 × *g* for 30 min to remove cellular debris and filtered through a 0.22-μm pore-size membrane (Millipore, MA, USA). Then the supernatants were concentrated by Vivaflow membrane (Sartorius stedim, Germany) according to the manufacture instruction. The concentration product was purified using gel filtration chromatography by sepharose 4FF and then ion exchange exclusion chromatography by DEAE-sepharose Fast Flow column (GE Healthcare, England). The purity of the EV71 virions was detected by high performance liquid chromatography (HPLC) (SHIMADZU, Japan) and quantified with the BCA protein assay kit (ThermoFisher, USA). EV71 particles were stored at −80°C.

### Construction of EV71 VP1 protein expression plasmid

QIAamp viral RNA mini Kit (Qiagen, Germantown, MD, USA) was used to extract EV71 RNA from infected Vero cell supernatants. Transcriptor High Fidelity cDNA Synthesis Kit (Roche, Indianapolis, IN, USA) was used for cDNA synthesis. The total 891 basepairs fragment of VP1 gene (Genebank No.HM038013) was amplified by polymerase chain reaction (PCR) using forward primer VP1(1–19)-F:CGC ***CATATG*** GGAGATAGGGTGGCAGATG and reverse primer VP1(874–891)-R:CCG ***CTCGAG*** AAGAGTGGTGATCGCTGT, the PCR product was digested with NdeI and XhoI and then cloned into pET30a vector (Novagen, Billerica, MA, USA) and named pET30a-VP1, the encoded fusion protein had a His-tag at the C-terminus.

### Purification and identification of recombninat VP1 protein and EV71 virions

BL21 (DE3) was transformed with pET30a-VP1 by the Ca^2+^ phosphate method [14]. Single clone was selected and inoculated into Luria Broth (LB) culture medium containing kanamycin (50 mg/ml). The culture medium was rotated at 37°C until the OD_600_ reached 0.6–0.7, culture medium was transplanted (1:1000 ratio) into 1.5-L Erlenmeyer flasks. Induction and purification of protein was according to the pET System Manual (11th edition, Novagen) using the Ni^2+^ affinity method. Further, potential endotoxin Lipopolysaccharide (LPS) in purified rVP1 protien solution was removed using ion exchange exclusion chromatography by Q Sepharose Fast Flow column (GE Healthcare, England), then the LPS levels were determined using the Limulus amebocyte lysate assay kit (ACC, USA ) according to manufacture instruction. Purified EV71 virions obtained from Beijing Institute of Biological Products Company Limited was analyzed by SDS-PAGE with 4-20% Mini-Protein TGX™ Gel (Bio-Red Cat No 4561093, USA) followed by staining with Coomassie brilliant blue. For western blot, proteins were transferred to polyvinylidene fluoride membranes (Bio-Rad, Hercules, CA, USA). Membranes were blocked for 1 h in 5% skim milk at room temperature followed by incubation with purified human recombinant monoclonal antibody to EV71 VP1, which was derived from a phage display liberary and converted to IgG form in bacuovirus/SF9 cell system [15]. Goat anti-human IgG conjugated to alkaline phosphatase was used as the second antibody. Signals were visualized with NBT/BCIP substrate (Roche, Germany).

### Preparation of immunogen EV71 rVP1-Chi and mice immunization

Immunogen rVP1-Chi was obtained by adding purified rVP1 40 μg or 160 μg to 1 ml 0.02% chitosan-phosphate buffer solution (PBS) (pH5.7) (Sigma, St. Louis, MO, USA). And high speed vortexing was used until the solution was homogenated. EV71 virions formulation was prepared as the rVP1. Female ICR mice (8 weeks old) were divided randomly into eight groups: (1) 40 μg rVP1 antigen (rVP1-40); (2) 160 μg rVP1 antigen (rVP1-160); (3) 40 μg rVP1 antigen with chitosan adjuvant (rVP1-40-Chi); (4) 160 μg rVP1 antigen with chitosan adjuvant (rVP1-160-Chi); (5) 40 μg purified EV71 virions antigen (Virion-40); (6) 40 μg purified EV71 virions antigen with chitosan adjuvant (Virion-40-Chi); (7) PBS (PBS); (8) PBS mixed with chitosan solution (PBS-Chi). Before oral immunization, mice were starved overnight and fed with sodium bicarbonate buffer to neutralize gastric acid. Eight mice per-group were immunized intragastrically on day 0, 7, 14 and 28. Blood samples were collected and centrifuged at 5000 × *g*, 4°C. Serum was inactivated at 56°C for 30 min and stored at −80°C until used for neutralization assays and measurement of serum IgG. Vaginal, respiratory tract, and small intestine secretions were collected by cold PBS washes (containing 0.5% saponin (w/v); 50 mM EDTA; and 0.1 mg/ml trypsin inhibitor) [16]. Fresh fecal pellets were collected on day 42, and immediately frozen at −80°C. Before analysis, 0.2 g feces was dissolved in 400 μl of cold PBS (containing 0.01% sodium azide and 1 mM EDTA), and the suspensions stirred and centrifuged at 9000 × *g* for 10 min. IgA in the supernatants was assayed by enzyme linked immunosorbent assay (ELISA) [17].

### Measurement of specific antibodies

VP1-specific IgG antibodies were measured by indirect ELISA by coating 96-well plates with EV71 virions (100 ng/well), overnight at 4°C. After blocking with 5% skim milk to avoid nonspecific binding, Serum diluted in 5% skim milk was added to each well and incubated for 1 h at 37°C. After washing with PBS contain 0.05% tween-20 (PBST, V/V), horse radish peroxidase (HRP) -conjugated goat anti-mouse IgG or anti-mouse IgA (Sigma, St. Louis, MO, USA) was added and incubated for 1 h at 37°C. 3,3’,3,5’-tetramethylbenzidine (TMB) was added as substrate, and absorbance was measured at 450 nm. The positive cut-off value was an optical density (OD) value 2.1-fold above normal negative control sample.

### Serum neutralization assay

The mice serum neutralizing antibodies against EV71 were analyzed using the cytopathic effect (CPE)-determination assay based on Vero cells. Briefly, mice sera from all groups were heat-inactivated at 56°C for 30 min, then were two-fold serially diluted from 1:4 to 1:256 in DMEM (2% FBS, 1% PS) and mixed with the same volume of 200 tissue culture infective dose 50 (TCID50) EV71 virus. After 2 h incubation at 37°C, 100 μl of virus-serum mixture was added to each well of the 96-well plates and followed by 100 μl of 2 × 10^5^/ml Vero cells. Every dilution of each serum sample was performed in quadruplicate. The plates were then incubated in a CO_2_ incubator at 37°C for 7 days. The neutralizing antibody titer was expressed as the maximum serum dilution at which CPE was not observed in all four wells. The serum dilution titer at start point of 1:4 was considered as the cut off value for sero-conversion determination.

### Splenocyte proliferation assay and cytokine production

Splenocytes were isolated from ICR mice from each vaccine group, which were sacrificed 6 weeks after primary inoculation, and cultured in 96-well plates (1 × 10^6^ cells per well) in 200 μl RPMI medium 1640 (10% FBS, 1% PS). 1 μg purified rVP1 protein was used as antigenic stimulant per well and concanavalin A (Con A) was served as positive control. Furthermore, to eliminate the cell activation due to the LPS contamination, splenocytes were also stimulated by nucleocapsid protein (NP) of Hantaan virus, which was expressed and purified the same way as rVP1. Plates were incubated at 37°C for 72 h. Culture supernatants from proliferating splenocytes were collected and the level of IFN-γ, IL-4, IL-5 and TGF-β were detected using mouse ELISA kits (R&D, Minneapolis, MN, USA). The concentration values of cytokines stimulated by rVP1 were all subtracted those stimulated by hantaan virus NP and were expressed as mean ± SD.

### Lethal EV71 challenge suckling mice

Because EV71 infection caused no apparent clinical symptoms in adult mice, viral challenge was performed using newborn mice. The 8-week-old female ICR mice received vaccination of rVP1-40, rVP1-40-Chi, rVP1-160, rVP1-160-Chi, Virion-40, Virion-40-Chi, PBS, and PBS-Chi as described above at day 0, 7, 14 and were allowed to mate. The mice received a booster 2 weeks later and neonatal mice were born at week 5 to week 6, then challenged intracerebrally with 10 μl (10^5^ PFU/ml) EV71 48–72 h after birth. Meanwhile, a challenge-free group was also included in this experiment in order to provide a basic survival background. The clinical symptoms and body weight changes of challenged suckling mice were recorded daily and the scores were graded as: health (0), inactivity and/or wasting (1), limb-shake weakness (2), hind limb paralysis (3), moribund and death (4).

### Statistical analysis

Results are expressed as mean ± SD. Significant differences between groups were analyzed using SPSS 11.5 (IBM, Chicago, IL, USA). *P* < 0.05 was considered statically significant. Significant differences between two means are presented as *P* < 0.05 (*) and *P* < 0.01 (**).

## Results

### Identification of EV71 virions and rVP1 protein

**Figure 1:**
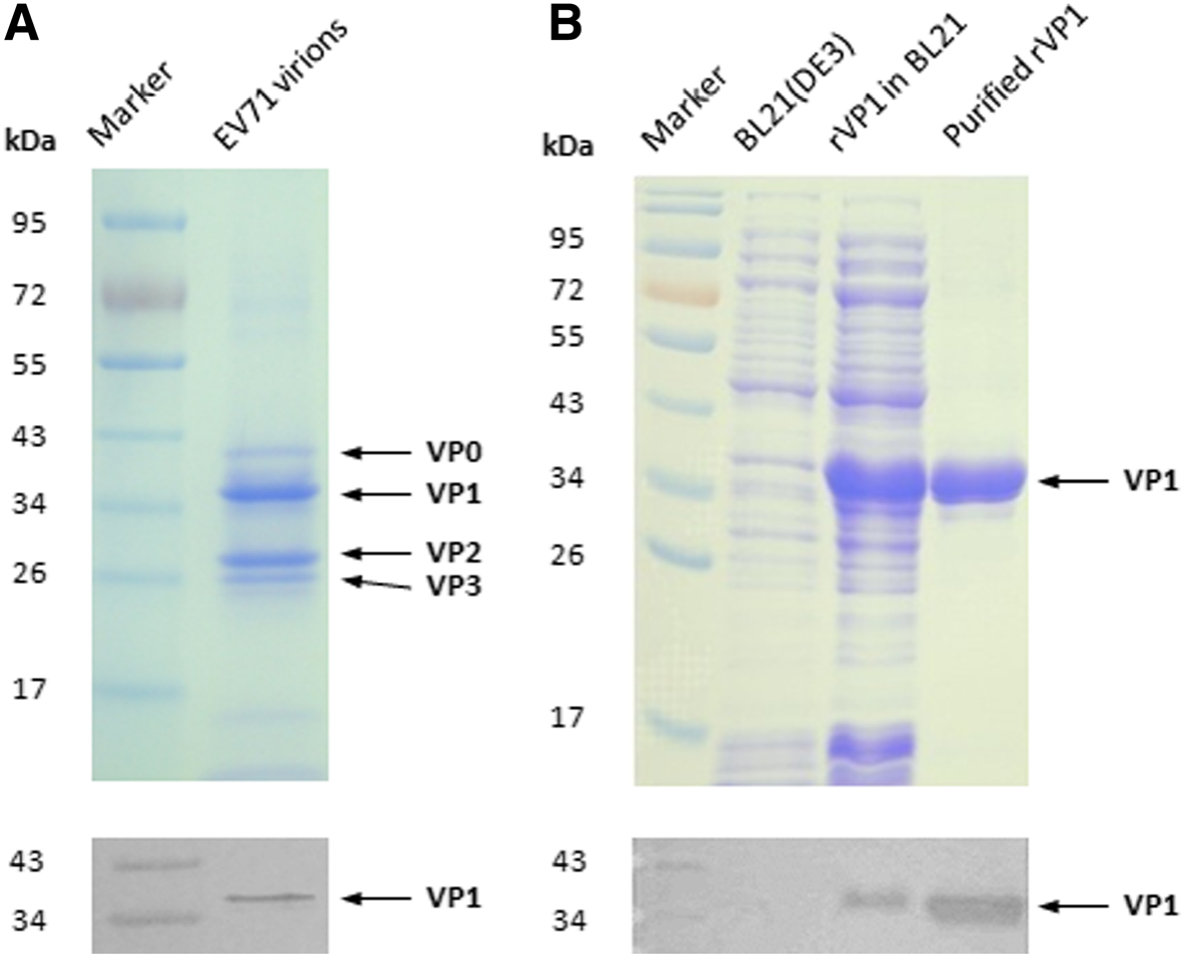
**Identification of purified EV71 virions and recombinant VP1.** Purified EV71 virions and rVP1 were identified by SDS-PAGE and Western blot analysis. **(A)** SDS-PAGE (up) and Western blot analysis (below) of purified EV71 virions. SDS-PAGE demonstrated the presence of VP0, VP1, VP2 and VP3, which was indicated by arrow bars and Western blot detected by anti-VP1 antibody showed specific reaction band of VP1 at molecular weight of 36 kDa. **(B)** SDS-PAGE (up) and Western blot analysis (below) of purified rVP1. Lane1, Marker. Lane2, BL21(DE3). Lane3, rVP1 expressed in BL21(DE3). Lane4, purified rVP1.

Purified EV71 virions and recombinant VP1 protein were characterized by SDS-PAGE and western blot analysis using anti-VP1 antibody. As shown in Figure 1, SDS-PAGE of EV71 virions demonstrated the presence of VP0, VP1, VP2 and VP3. The molecular weight of EV71 rVP1 was about 36 kDa, which was similar to VP1 of authentic virions. Western blot analysis detected by specific antibody further confirmed the identity of rVP1, showing that both virions and rVP1 presented similar reaction bands at molecular weight of 36 kDa. The purity was above 95% for EV71 virions and rVP1 as assessed by HPLC. And the concentration of rVP1 protein was finally adjusted to 2 mg/ml with BCA kit (Pierces, USA), in which the LPS level was detected below 1500 EU/mg with the kit mentioned above.

### Recombinant VP1 elicits specific IgG and neutralization antibodies

**Figure 2:**
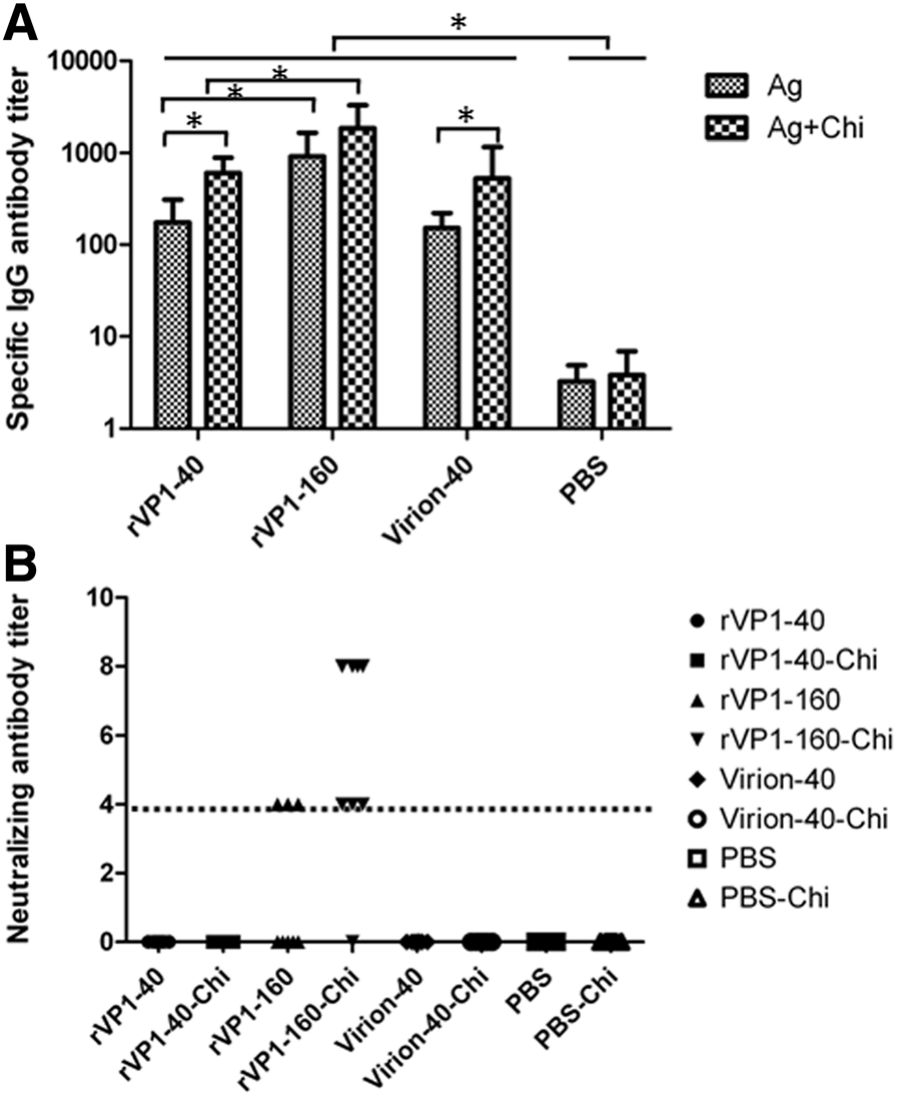
**Titer profiles of total IgG and neutralizing antibody. (A)** Titers of total anti-EV71 IgG. Data were expressed as mean ± SD. (*P < 0.05, ** *P* < 0.01). **(B)** Neutralization titers. Groups of female ICR mice (n = 8) were Immunized orally on days 0,7,14 and 28 with different formulation of vaccine, the dot line indicate the initial dilution of mice serum.

To determine whether the recombinant VP1 protein was capable of eliciting anti-EV71 antibodies, serum samples of eight groups of female ICR mice were collected six weeks after primary immunization for antibody assays. As is shown in Figure 2A, in comparison to PBS control group, specific IgG antibodies could be detected in all vaccine-immunized groups after three times of boosts when using EV71 virions as coating antigen of ELISA. Groups of 160 μg rVP1 or rVP1-Chi induced higher levels of specific IgG antibody titers (1:1600 or 1:3200) than those received 40 μg rVP1 or rVP1-Chi (1:200 or 1:800) (P < 0.05) (Figure 2A) and the chitosan could significantly enhance the titer of IgG antibodies (P < 0.05) (Figure 2A) when giving the same dose of antigen. No significant differences between rVP1 and EV71 virions groups were observed.

The ability of serum antibodies to neutralize live EV71 virus in vitro was examined by neutralization assay based on Vero cell. Results showed that mice immunized with rVP1-160 or rVP1-160-Chi developed neutralizing antibody titers that could effectively against homologous EV71 strain at dilutions of 1:4 or 1:8. The sera conversion rate for neutralization antibodies was 37.5% (3/8) in rVP1-160 group and 87.5% (7/8 in rVP1-160-Chi group, respectively (Figure 2B). However, as the specific IgG titers were lower than rVP1-160 μg group, the neutralizing antibodies were not detected (lower than 1:4) in the groups of rVP1-40, rVP1-40-Chi, virion-40 and virion-40-Chi indicated that higher dose of rVP1 mucosal immunization may be required for efficiency neutralizing antibody production and specific T cell responses.

### Recombinant VP1 induces specific mucosal IgA antibodies

**Figure 3:**
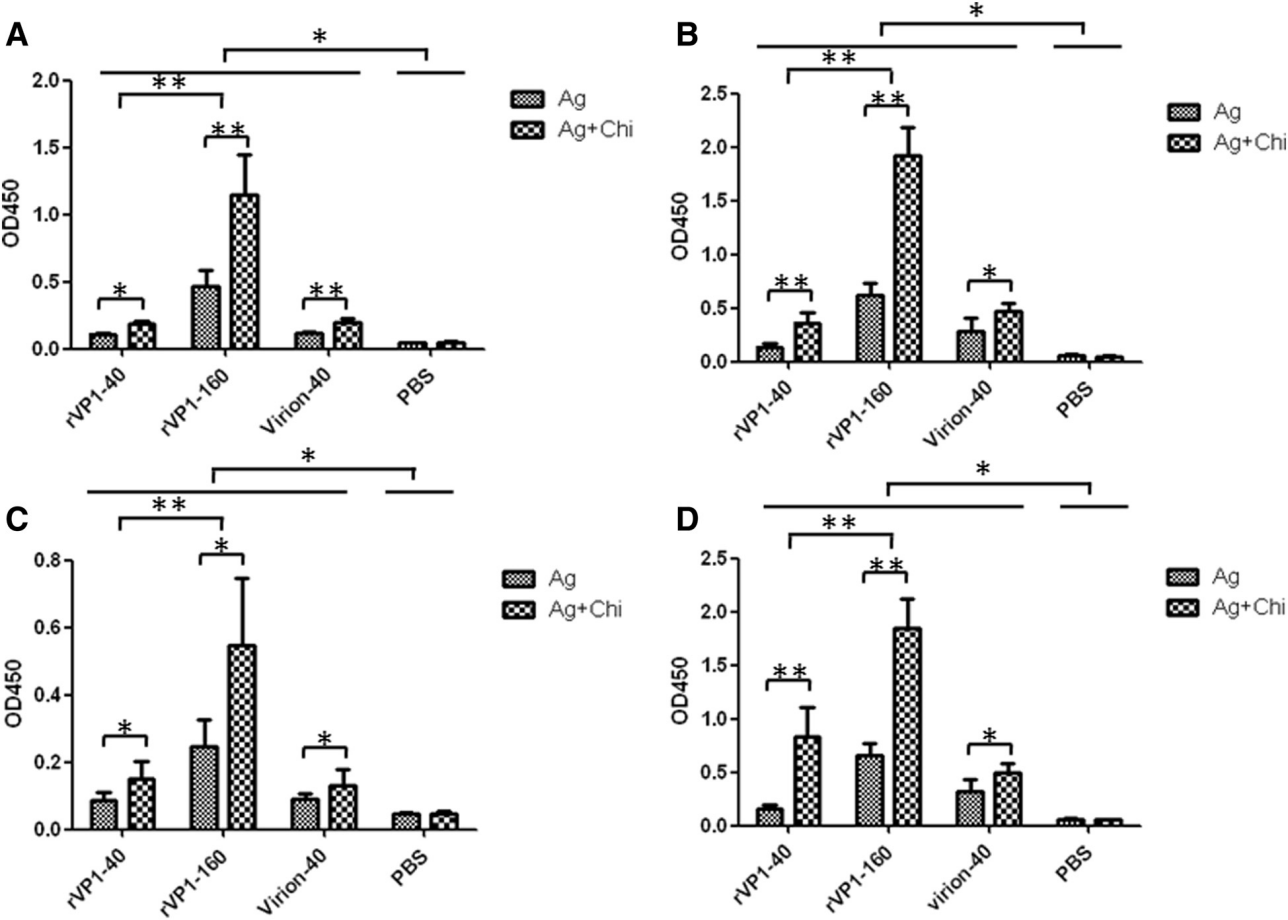
**Detection of EV71 specific mucosal IgA antibody.** Secretions of vaginal, intestinal, respiratory tract and fresh fecal pellets were collected on day 42 and immediately frozen at −80°C. Titers of specific IgA antibody in: **(A)** Intestine (diluted at 1:10); **(B)** Stool homogenates (diluted at 1:4); **(C)** Respiratory tract (diluted at 1:2); **(D)** Vaginal secretions (diluted at 1:2) were detected by indirect ELISA. Data were expressed as mean OD.450 value ± SD. (* *P* < 0.05, ** *P* < 0.01).

To test whether EV71 rVP1 can elicit mucosal immune responses, samples in Vaginal, respiratory tract, small intestine secretions and feces were collected on day 42 after the first inoculation and mucosal IgA antibody titers were measured by ELISA. Results showed that mice immunized with either EV71 rVP1 or virions displayed apparent mucosal responses as compared with PBS negative control groups (Figure 3). Furthermore, the mice immunized with 160 μg rVP1 significantly (P < 0.05) induced higher mucosal IgA levels than the mice immunized with 40 μg rVP1. Again the mucosal responses elicited by mice immunized with chitosan associated rVP1 or virions were also significantly higher (P < 0.05) than those of mice immunized with non-associated antigens when giving the same dose. No statistical difference was observed between groups of recombinant VP1 protein and virions.

### Cytokine production profiles

**Figure 4:**
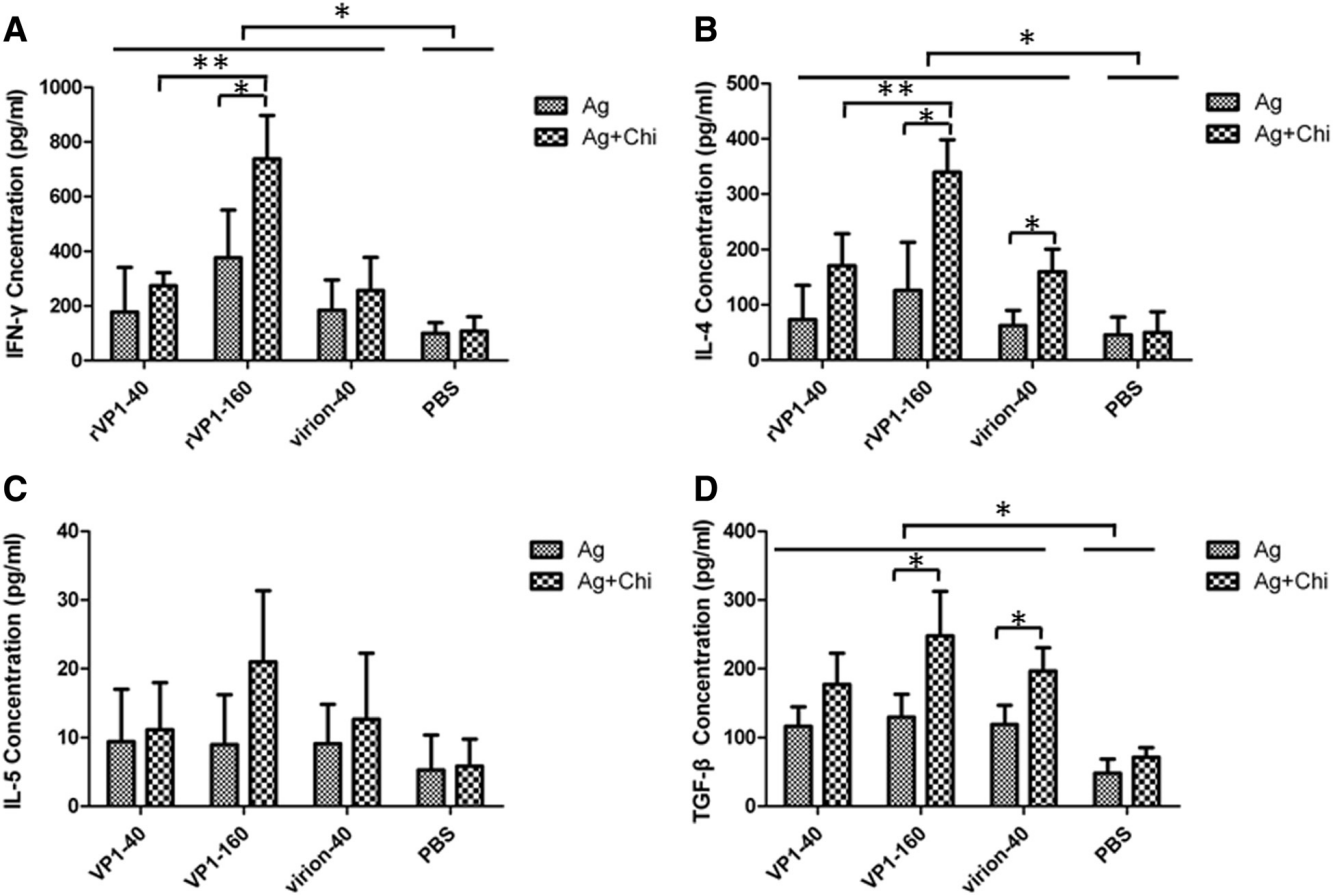
**Cytokine production profiles.** Splenocytes from groups of immunized mice were stimulated with recombinant VP1 protein for 72 h. Concentrations of **(A)** IFN-γ. **(B)** IL-4. **(C)** IL-5. **(D)** TGF-β in the culture supernatants were determined by ELISA. The concentration values of cytokines were expressed as mean ± SD. (* *P* < 0.05, ** *P* < 0.01).

Profiles of cytokine production by splenocytes were studied as indicators of the polarization of Th1/Th2 immune response. The IFN-γ, IL-4, IL-5 and TGF-β cytokines in the supernatants of splenocytes after stimulation with rVP1 were investigated by ELISA (Figure 4). Groups of mice immunized with rVP1 or virions induced considerable levels of IFN-γ compared with mice immunized with PBS (P < 0.05) (Figure 4A), confirming the induction of Th1 cellular immune responses by oral inoculation. Splenocytes of mice immunized with high-dose antigen produced significantly higher levels of IFN-γ than mice vaccinated with low-dose antigen (P < 0.05) and the chitosan enhanced induction of the Th1 cellular immune response when high-dose of rVP1 administrated (P < 0.05). No significant differences were observed between groups immunized with 40 μg rVP1 protein and virions. As for cytokine IL-4 detection (Figure 4B), which is the indicator of Th2 immune responses, spleen cells of mice immunized with recombinant VP1 protein or virions produced higher level of IL-4 than PBS control groups (P < 0.05). Splenocytes derived from the rVP1-160-Chi group produced the highest level of IL-4. These results demonstrated that rVP1 antigen could induce a mixed Th1 and Th2 immune response in the ICR mouse model. Splenocytes of mice immunized with vaccine groups including rVP1-40, rVP1-40-Chi, rVP1-160, rVP1-160-Chi, virion-40 and virion-40-Chi produced higher levels of TGF-β than PBS group, and the chitosan could enhance induction of TGF-β when giving 160 μg of rVP1 (P < 0.05) (Figure 4D). The production of IL-5 was low overall and no significant difference between groups was detected (Figure 4C).

### Recombinant VP1 protects suckling ICR mice against lethal virus challenge

**Figure 5:**
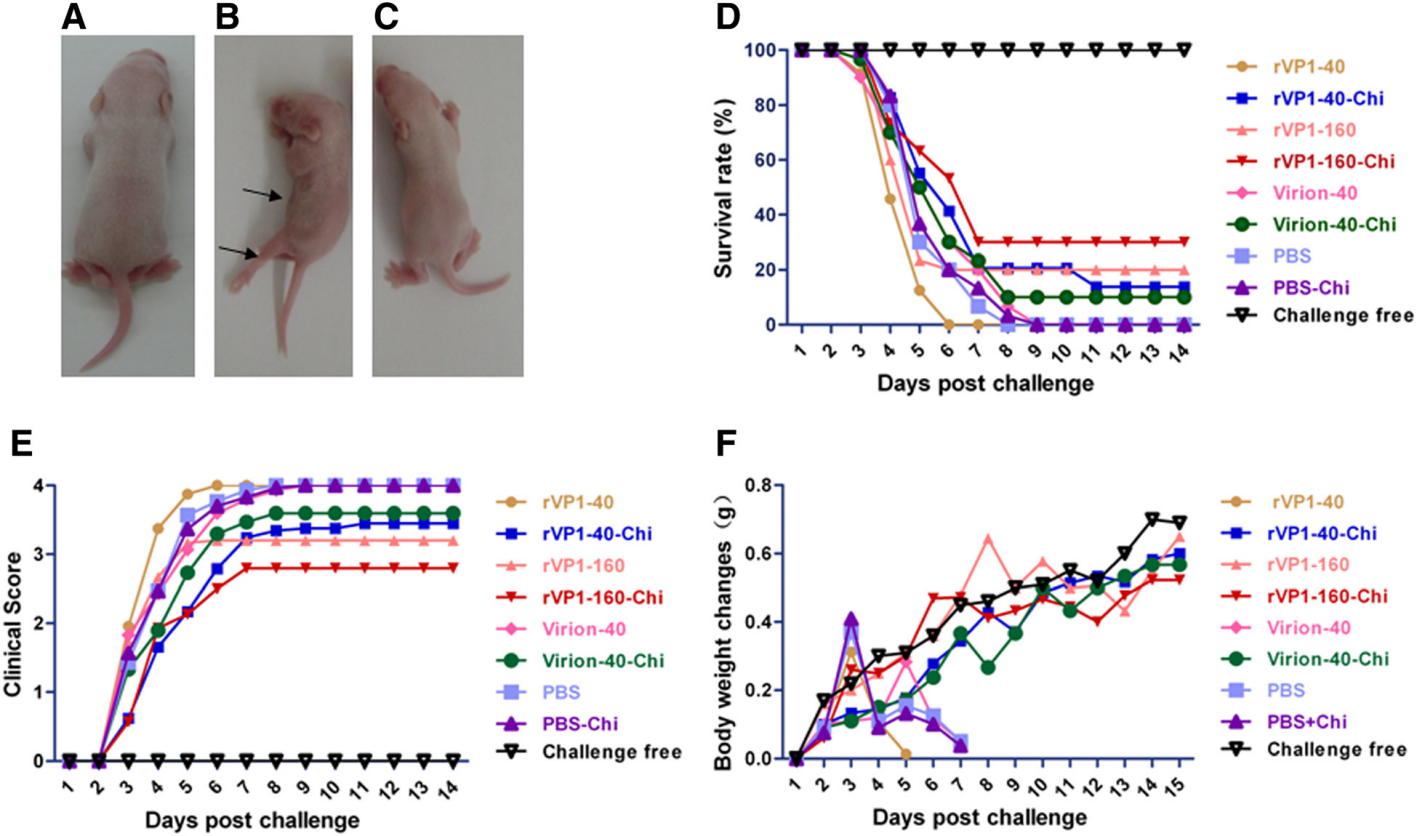
**Lethal EV71 challenge of newborns from immunized female mice.** Two-day-old pups from immunized female mice were intracerebrally inoculated with EV71. **(A)** Unchallenged suckling mouse **(B)** representative mouse in the PBS group with limb paralysis and wasting (arrows) at 3 days post-infection with EV71 virus; **(C)** protected mouse challenged with EV71 virus; **(D)** survival rate after viral challenge of suckling mice from mothers immunized with different oral vaccines; **(E)** mean clinical scores for clinical symptoms of pups from female mice immunized with different vaccines; **(F)** weight changes in suckling ICR mice after EV71 challenge. Body weights were recorded for all surviving mice daily until 14 days post-infection.

Protection against lethal EV71 infection by the passively transferred maternal antibody from immunized dams was evaluated in newborn ICR mice. 2 days after challenge, un-protected neonatal mice started to show clinical symptoms, such as wasting, hind-limb paralysis and eventually deaths were occurred within 2 weeks (Figure 5A,B,C,E,F). At virus challenge dose of 10^3^ PFU, the protection in newborns was markedly different according to the vaccine preparation received by their mothers (Figure 5D). The suckling mice born to dams that received rVP1-160-Chi, in which group the neutralizing antibody titers were about 1:8, showed a protective efficacy of 30.0% (9/30). Protection efficacy against lethal infection was 13.8% (4/29) for rVP1-40-Chi, 20% (6/30) for rVP1-160 (neutralizing antibody titers were 1:4) and 10% (3/30) for the group that received Virion-40-Chi. Groups that received rVP1-40 (0/24), Virion-40 (0/30), PBS (0/30) or PBS-Chi (0/30) failed to provide protection for newborn mice and all challenged mice died within 14 days (Figure 5D). Meanwhile, all newborn ICR mice in challenge free group survived.

## Discussion

The human pathogen of EV71 causes the infectious disease HFMD mostly in children. The virus initiates children infection via the mucosal surfaces and therefore the mucosal immunization could be a promising way to achieve protection against the virus infection in human. In this study, the efficacy and protective functions of oral immunization with chitosan and recombinant EV71 VP1 formulations were analyzed and evaluated in ICR mouse model with lethal challenge of EV71, and the results presented that chitosan help EV71 rVP1 induced better VP1-specific IgA antibodies in intestine, feces, vagina, and respiratory tract, and systemic specific IgG and neutralization antibodies in humoral immune responses as well as the Th1 and Th2 cellular immune responses.

The mucosal surfaces of the gastrointestinal, genitourinary, and respiratory tracts are the primary entry sites of most pathogens. The induction of effective mucosal immunity could greatly reduce the infection rate and morbidity. Thus, mucosal immunization, especially by the oral route, has recently attracted much interest. IgA is thought to protect the host against pathogens that infect mucosal surfaces and those that cause systemic disease after entry via a mucosal surface [18]. In our study, rVP1 oral vaccine induced specific IgA antibody immune response on the mucosal surface. IgA antibody was detected in feces and washes of small intestine, and also detected in the mucosal secretion of vagina and respiratory tract. The reason might be the different mucosal surfaces are interconnected via circulating lymphocytes, recognized as the common mucosal immune system [19], which lead immunization at one mucosal inductive site to an immune response at a remote mucosal effective site. Except the mucosal IgA antibody production, we also find the cytokine TGF-β related to IgA production was significantly elevated from rVP1 160 μg with chitosan, suggest that the involvement of TGF-β in the mucosal IgA antibody response in the mice immunized with rVP1. TGF-β was produced mainly by the Th3 type lymphocytes, the production of cytokine TGF-β indicated the Th3 type immune responses involved in the rVP1 mucosal immune responses. However, as previously reported [17,20], no significant changes in IL-5 secretion was observed in our study.

Oral immunization is complicated by antigen degradation in the gastrointestinal tract and low uptake into M cells in Peyer's Patches. Induction of immune responses following oral immunization is usually dependent on the co-administration of appropriate adjuvant that can initiate and support the transition from innate to adaptive immunity. In this study, we chose chitosan as a mimic adjuvant or a component in vaccine delivery system to enhance the immunogenicity of the rVP1, our results showed that chitosan based delivery and adjuvant formulations were effective in production of specific humoral and cellular immune responses and protection of ICR mice form EV71 challenge. Chitosan is a naturally occurring polysaccharide material that is biodegradable and non-toxic. Due to the function of penetration and uptake enhancement and antigen protection, chitosan has been clearly demonstrated as a promising candidate for mucosal vaccine delivery system in mice [21,22], it has also been reported to have immune stimulating activities such as increasing the accumulation and activation of macrophages, promoting resistance to infections by enhancing innate and cytotoxic T lyphocyte (CTL) immune responses [23]. However, the working mechanisms of chitosan in vaccine delivery system still need to be further understood.

In this study, we also find that both rVP1-160 and rVP1-160-Chi groups developed high titers of specific antibodies and effective neutralizing antibodies, but the neutralizing antibodies were not detectable for groups of rVP1-40, rVP1-40-Chi, virion-40 and virion-40-Chi. However, both rVP1 doses of 160 μg and 40 μg with chitosan showed certain level of protection rate in EV71 challenged ICR mice although the protection rates were not satisfied. The in vivo functions of the neutralizing antibodies for rVP1-40-Chi and virion-40-Chi groups may not be sensitively measured by the micro-neutralizing assay in vitro. On another hand, the protective function in vivo may be not only offered by the neutralizing antibodies and the cellular immune responses. The cell-mediated immune responses were also detected after oral immunization. Administration of rVP1 with chitosan induced increases in the levels of the secreted Th1 cytokine IFN-γ and Th2 cytokine IL-4 in immunized mice indicating that the EV71 rVP1 oral vaccine might have triggered Th1 and Th2 immune responses, which are involved in cellular-mediated immunity. Innate immune responses might also be important in protection of newborn mice against EV71 challenge. This might explain of the differences in the *in vitro* and *in vivo* experimental results. Furthermore, virions usually induced better humoral and cellular immune response in intramuscular injection type of vaccine. But in this study, the virions at dose 40 μg could not induce better antibody titers and the T cell responses as the rVP1-40 group. The reason might be the digestion effect of gastric acid and protease, the antigen achieved the effective target sites in small intestine was limited. The higher dose virus particles may stimulate better immune response.

## Conclusions

In conclusion, oral delivery of rVP1 in formulation with chitosan induced systemic humoral and multi-mucosal immune responses, as neutralization antibodies blocked EV71 from infecting host cells *in vitro* and maternally transferred antibodies or cellular immunity protected neonatal mice against EV71 challenge *in vivo*. Our results indicate that oral immunization with rVP1 in formulation with chitosan is effective in inducing broad-spectrum immune responses and may be a promising subunit vaccine candidate for the prevention of EV71 infection, a non-invasive oral vaccination strategy may be an attractive alternative for developing countries, in particular acceptable to children and easier for vaccination in developing countries.

## Competing interests

The authors declare that they have no competing interests.

## Authors’ contributions

ZF and ZS performed most of the experiments and involved in manuscript preparation. HC and LA participated in mice immunization and detection of humoral immune responses. ZQ and LC were involved in virus infection and purification. WW and LL participated in the detection of cellular immune responses. LX provided EV71 and gave advices for the project. LM coordinated laboratory manipulation and edited the manuscript. LD is the project leader and was involved in project design, manipulation, data analysis and finalization of the manuscript. All authors read and approved the final manuscript.
